# From in vitro to in vivo: hypoxia attenuates replicative senescence and preserves therapeutic activity in long-term passaged human umbilical cord-derived mesenchymal stem cells

**DOI:** 10.1186/s40659-026-00703-z

**Published:** 2026-06-02

**Authors:** Chenxi Jia, Ze Lin, Tianrui Zhang, Lingbin He, Yueyang Li, Jianhong Li, Xinyi Li, Yuan Zhang, Bozhi Cai, Muping Zou, Libiao Wu, Xutao Zhao, Chunhong Ren, Xin Zhang

**Affiliations:** 1 Laboratory of Molecular Cardiology, The First Affiliated Hospital of Shantou University Medical College, 57Th Changping Road, Shantou, 515041 Guangdong Province People’s Republic of China; 2 Shantou University Medical College, Shantou, Guangdong Province People’s Republic of China; 3 Medical Department, Hubei Enshi College, Enshi, Hubei Province People’s Republic of China; 4 Clinical Research Center, The First Affiliated Hospital of Shantou University Medical College, Shantou, Guangdong Province People’s Republic of China; 5 Guang’anmen Hospital Jinan Branch, Jinan Municipal Hospital of Traditional Chinese Medicine, Jinan, 250012 Shandong Province People’s Republic of China; 6 International Medical Service Center, The First Affiliated Hospital of Shantou University Medical College, Shantou, Guangdong Province People’s Republic of China

**Keywords:** Oxygen concentration, Human umbilical cord, Mesenchymal stem cells, Cellular aging, Continuous passaging

## Abstract

**Background:**

Mesenchymal stem cells (MSCs) hold great potential for regenerative medicine and tissue engineering. However, their clinical use is limited by low in vivo availability and rapid senescence during ex vivo expansion, posing a major challenge for scaled production. Growing evidence indicates that microenvironmental oxygen tension critically regulates MSCs fate. This study aims to systematically evaluate how different oxygen tensions modulate MSCs aging and function, providing evidence to optimize MSCs expansion protocols for clinical applications.

**Methods:**

Human umbilical cord derived-mesenchymal stem cells (hUC-MSCs) were maintained under normoxic (20%O_2_) and hypoxic (5%O_2_) conditions through continuous passaging until senescence occurred. In vitro analysis systematically compared proliferation, cell cycle distribution, apoptosis, and senescence biomarkers across serially expanded populations from both oxygen concentrations. For in vivo evaluations, a type 2 diabetic nephropathy mouse model was used to investigate therapeutic efficacy. Mice received systemic transplantation of hUC-MSCs from each group, and renal functional recovery along with histopathological changes were assessed.

**Results:**

Cells cultured under both 20%O_2_ (normoxia) and 5%O_2_ (hypoxia) stably expressed MSC-specific surface markers throughout serial passages. Hypoxia-cultured cells retained enhanced adipogenic and osteogenic differentiation potential, along with superior proliferative capacity and growth factor secretion, through at least 16 passages, consistently outperforming normoxic counterparts. Hypoxic conditions also attenuated replicative senescence, suppressed DNA damage, and reduced secretion of SASP-associated pro-inflammatory factors (including IL-6, IL-8, and TGF-β), while decreasing apoptosis. In a diabetic nephropathy mouse model, P16 MSCs cultured under hypoxia still significantly improved renal function and attenuated fibrosis post-transplantation, as demonstrated by reduced 24-h urinary protein, serum creatinine, and blood urea nitrogen levels compared to the normoxic MSCs group.

**Conclusion:**

We provide the first in vivo evidence that hypoxic culture conditions enables high-passage hUC-MSCs(P16) to retain therapeutic potency in diabetic nephropathy. Our findings demonstrate that hypoxia effectively mitigates replicative senescence through suppression of oxidative damage and SASP secretion, thereby preserving the proliferative capacity and paracrine repair functions of long-term cultured cells.

**Graphical abstract:**

**Figure:**
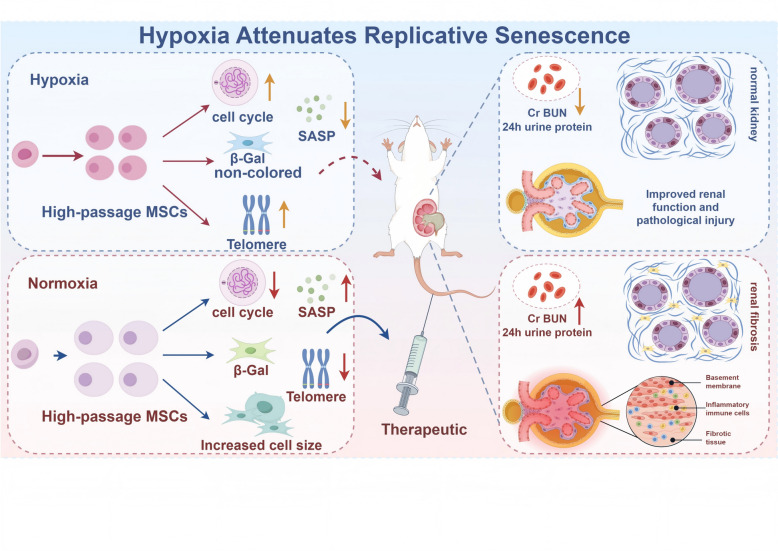

**Supplementary Information:**

The online version contains supplementary material available at 10.1186/s40659-026-00703-z.

## Background

Human mesenchymal stem cells, known for their self-renewal and multipotent capacities, have been successfully isolated and expanded from a variety of tissues including bone marrow, adipose tissue, umbilical cord tissue, dental pulp, and placental tissues [1–3]. Moreover, Mesenchymal stem cells (MSCs) also exhibit potent immunomodulatory functions [4], autocrine/paracrine activities [5], and low immunogenicity, thereby attracted significant interest in the fields of regenerative medicine and tissue engineering. Mounting evidence has made MSCs the most promising alternative to conventional treatments for autoimmune diseases, degenerative diseases, and ageing related diseases. Achieving optimal therapeutic outcomes requires the administration of sufficient quantities of high-quality MSCs. Since their natural abundance in donor tissues is low, in vitro expansion becomes a necessary step before clinical application. However, during large-scale and long-term serial passaging, MSCs reach the Hayflick limit, entering a state of replicative senescence, accompanied by markedly reduced proliferation eventually leading to growth arrest [6]. Meanwhile, MSCs undergo morphological and functional alterations such as cell enlargement and flattening, loss of differentiation potential, diminished secretory function, and reduced therapeutic efficacy [7–11]. Therefore, it is of utmost importance to develop and optimize manufacturing protocols that not only supports large-scale expansion of MSCs but also effectively delay cellular senescence while preserving their functional biological properties.

While the importance of nutrients, growth factors, temperature, and pH in cell culture is well recognized and routinely controlled in practice, the role of oxygen tension has received far less attention [12]. Currently, ambient air conditions—with oxygen concentrations around 21% or slightly lower in standard CO_2_ incubators (− 19%)—remain the standard for in vitro MSCs expansion and are typically referred to as “normoxia”. However, in vivo oxygen levels in most organs or tissues range from 2 to 10%, and can even fall below 1% in certain microenvironments such as the kidney, thymus, or solid tumor [13–15]. Thus, while often labeled “normoxic”, conventional cell culture conditions actually represent a non-physiological hyperoxic state relative to the physiologically hypoxic conditions in native tissue niches. Despite this significant discrepancy, the difference between in vitro and in vivo oxygen environments is frequently overlooked.

Given that elevated oxygen tension can induce oxidative stress, leading to DNA damage and cell senescence [16], it has been postulated that maintaining MSCs under hypoxic conditions may improve their overall quality. Consequently, a growing number of studies have explored the influence of low O_2_ tension on the biological properties of MSCs. Although outcomes vary across studies, largely due to differences in MSCs tissue sources, oxygen concentrations (ranging from 1 to 10%), and culture conditions, most reports have consistently observed beneficial effects of hypoxic culture on MSCs survival, proliferation, migration, and differentiation potential. In particular, multiple lines of data suggested that hypoxic condition can delay cellular senescence and reduce the expression of senescence-associated markers [17–22].

However, most existing studies have focused on short-term or transient hypoxic exposure, while comparative studies examining the effects of prolonged hypoxia versus normoxia on MSCs senescence and biological functionality in vitro remain limited. More importantly, robust in vivo evidence validating the functional relevance of these hypoxic adaptations, particularly whether the therapeutic efficacy of MSCs is maintained after long-term serial passaging under hypoxia is still absent [23, 24].

This study systematically evaluates the effects of sustained hypoxia on Human umbilical cord derived-mesenchymal stem cells (hUC-MSCs) both in vitro and in vivo. Low-passage hUC-MSCs were maintained under normoxic (20%O_2_) or hypoxic (5%O_2_) conditions and subjected to serial passaging. The anti-senescence benefits and functional alterations resulting from hypoxic conditioning were assessed at multiple passage time points. Most significantly, to directly assess the physiological relevance, we established a type 2 diabetic nephropathy (DN) mouse model to evaluate and compare the therapeutic efficacy of cells expanded under normoxic versus hypoxic conditions across successive passages.

## Methods

### Expansion and characterization of hUC-MSCs

Primary hUC-MSCs (P0 cells) were obtained from Fengzekang Biopharmaceutical Co., Ltd. (Shenzhen,China) and cultured at 37 °C with 95% humidity, 5%CO_2_ under either 5%O_2_ (hypoxic) or 20%O_2_ (normoxic) conditions. Cells were harvested at 80–90% confluency, with experiments conducted on passages4-16. Multilineage differentiation potential was confirmed through Oil Red O staining for adipogenesis and Alizarin Red staining for osteogenesis using Thermo Fisher staining kits. Specific surface marker expression was analyzed by flow cytometry (BD Accuri C6), assessing MSCs-positive markers (CD29, CD44, CD73, CD90, CD105) and exclusion markers (HLA-DR, CD31, CD34) with FlowJo software (BD Biosciences).

### Cell proliferation assay

Passage4 and passage16 cells maintained under hypoxic or normoxic conditions were plated at a density of 500 cells/cm^2^ in a 96-well plate. Cell proliferation assay were conducted everyday, day 0 through day 6 culture. After diluting the CCK-8 reagent with hUC-MSCs culture medium at a ratio of 1:10, 100 μL of the diluted CCK-8 solution was added to each well in the experimental groups and blank group. The plate was then incubated at 37 °C for 1 h in dark, then 96 well plate was measured the absorbance at 450 nm using SYNERGY H1 full-wave length microplate reader (BioTek, USA) and OD values were obtained, and the data were analyzed with the CHS3.8 software.

Starting from Passage4 and continuing every four passages until Passage16, cells maintained under hypoxic or normoxic conditions were trypsinized and harvested, followed by washing with PBS. The cells were then resuspended in 300 μL of PBS, and 900 μL of 95% ethanol was added for fixation for 12 h, followed by PBS washing. The cells were then resuspended in 100 μL of PBS and incubated at room temperature with 2 μL of Ki67 (BioLegend, USA) solution for 15 min. Cell proliferation was assessed using a flow cytometer (BD Accuri C6), and the data were analyzed using FlowJo software (BD, USA). The fluorescence intensity of the cells was evaluated (BD FACSVerse™) and analyzed using BD FACSuite™ software.

### Cell viability analysis

Cells were trypsinized and harvested, followed by PBS washing. Then, the cells were resuspended in 500 μL of binding buffer and then incubated at room temperature with 3 μL of Annexin V-Alexa Fluor 647 and 5 μL of 7-aminoactinomycin D (7-AAD) solution for 15 min (provided by 4A Biotech, Beijing, China). Cell apoptosis was evaluated by flow cytometry (BD Accuri C6), and the data were analyzed using FlowJo software (BD, USA). The fluorescence intensity of the cells was evaluated (BD FACSVerse™), and were analyzed using the BD FACSuite™ software.

### Cell cycle analysis by flow cytometry

Cells were trypsinized and harvested, followed by PBS washing. Then, the cells were resuspended in 300 μL of PBS, and 900 μL of 95% ethanol was added dropwise to fix them for 12 h, followed by PBS washing. Then, 400 μL of staining buffer, 15 μL of propidium iodide staining solution (25X), and 4 μL of RNase A (2.5 mg/mL) were added to each sample and incubated at 37 °C in the dark for 30 min (provided by 4A Biotech, Beijing, China). Cell cycle analysis was performed by flow cytometry (BD Accuri C6), and Modfit LT™ was used to assess the distribution of hUC-MSCs across different cell cycle phases.

### Reactive oxygen species detection by DCFH-DA fluorescent probe

DCFH-DA (Sigma, USA) was diluted to a concentration of 5 μM using DMEM/F12 basal medium, and the solution was then mixed evenly and added to cells maintained under hypoxic or normoxic conditions. The cells were incubated at 37 °C for 20 min, avoiding light throughout the process. Reactive oxygen species (ROS) were detected using a flow cytometer (BD Accuri C6), and the data were analyzed using FlowJo software (BD, USA). The fluorescence intensity of the cells was evaluated (BD FACSVerse™) and analyzed using BD FACSuite™ software.

### RNA extraction and real-time PCR

Cells maintained under different oxygen concentrations and passage numbers were collected. RNA purification was conducted using the EasyPure® RNA Kit (TransGen, Beijing, China) in accordance with the manufacturer's instructions. Following this, genomic DNA was removed, and cDNA was synthesized using the Evo M-MLV Reverse Transcription Premix Kit (AGbio, China). Subsequently, quantitative real-time polymerase chain reaction (ABI StepOne Plus) was performed on the synthesized cDNA using SYBR Green Master Mix (AGbio, China) and the primers detailed in Table 1. Following the manufacturer's instructions, the probe-based qPCR kit (tjs, CAT#17–001-htert) was used to detect the core telomerase gene *TERT*. All analyses and measurements were conducted using the LightCycler® 480 II Real-Time PCR Detection System (Roche).

**Table 1:** Primer pairs for each gene

Gene	Primer sequence	Product size (bp)
*LAMNB1*	F:5'-GAGAGCAACATGAGCCAAGTG-3'	129
	R:5'-GTTCTTCCCTGGCACTGTTGAC-3'	
*P16*	F:5'-GAGCAGCATGGAGCCTTC-3'	116
	R:5'-CGTAACTATTCCGTGCGTTC-3'	
*P21*	F:5'-GCCATTACCCCATCACACT-3'	140
	R:5'-ACCGAGGCACTCAGAGGAG-3'	

### Telomere length analysis

Genomic DNA was extracted from cells, and telomere length was assessed using the Telomere Length Quantification qPCR Kit (tjs, CAT#14-001rTL) according to the manufacturer's protocol. Relative telomere length (T/S ratio) was calculated by comparing the copy number of telomeric repeat sequences to that of a single-copy reference gene, with normalization performed using primers targeting a 78 bp region on human chromosome 11. The T/S ratio is a widely accepted approach for relative telomere length quantification, in which T denotes the telomeric repeat copy number and S refers to the copy number of a stably expressed single-copy reference gene. This ratio normalizes variations in DNA input and amplification efficiency across samples, thereby allowing reliable and consistent comparison of relative telomere length among different cell passage groups [25]. All qPCR reactions were run on the CFX 96 Touch™ Real-Time PCR Detection System (Bio-Rad).

### Enzyme-linked immunosorbent assays

P4, P10, and P16 hUC-MSCs maintained under hypoxic or normoxic conditions were seeded in a 6-well plate at 20,000 cells/cm^2^. Following cell attachment, the cells were washed with PBS, and incubated with 1 mL phenol red-free DMEM/F12 basal medium (Procell, China) for 24 h. Supernatants were then collected for analysis. Secreted levels of FGF, VEGF, IL-6, IL-8, and TGFβ were quantified using ELISA kit (4A Biotech, Beijing, China). DNA damage biomarker 8-OHdG was measured with a separate ELISA kit (spbio, Wuhan, China).

### Senescence associated β-galactosidase (SA-β-gal) staining

Passage16 hUC-MSCs maintained under hypoxic or normoxic conditions were seeded in a 6-well plate at 20,000 cells/cm^2^. Upon reaching approximately 80% confluency, cells were fixed for 15 min, washed thrice with PBS, and incubated overnight in a 37 °C with 1 mL of β-galactosidase staining solution (Beyotime Biotechnology, Shanghai, China). Stained cells were Imaged using an inverted phase contrast microscope (Leica, Germany).

### Mouse model of diabetic nephropathy and hUC-MSCs treatment

All procedures were approved by the Medical Ethics Committee of the First Affiliated Hospital of Shantou University (ethical approval number: SUMC2021-267), Male ICR mice (4-week-old, n = 60; Beijing Vital River Laboratory Animal Technology) were housed under specific pathogen-free (SPF) conditions (24–26 °C, 12-h light/dark cycle) at the Experimental Animal Center of Shantou Medical College, each mice considered an experimental unit. The mice were randomly assigned to the following groups: control group, DN group, HypoxiaP4 treatment group, NormoxiaP4 treatment group, HypoxiaP16 treatment group, and NormoxiaP16 treatment group. We sample size (n = 6/group) was determined based on animal welfare considerations and in line with a previous study [26]. Mice that died unexpected during the extended modeling period for diabetic nephropathy, which prevented data collection, were excluded from the analysis. A final sample size of n = 4 per group was ensured to maintain data accuracy and consistency. After 1 week of normal diet, the model mice received high-fat diet (HFD, D12492 60% kcal% fat) for 3 weeks. Following overnight fasting, streptozotocin (STZ; 40 mg/kg) was administered intraperitoneally for 4 consecutive days. Diabetic mice (blood glucose ≥ 16.7 mmol/L at 1 week post-STZ) continued HFD for 6 more weeks. Sustained hyperglycemia with urinary protein levels ≥ 20 mg/kg significantly exceeding control group levels, along with accompanying renal pathological changes, is defined as DN models. The treatment group mice received weekly tail vein injections of hUC-MSCs (2 × 10^5^ cells/200 μL PBS) for a duration of 4 weeks. To avoid potential confounding effects from the cell injection, an equivalent volume of PBS was administered to the control group. The study was performed by two investigators: the first investigator performed the random allocation of mice to treatment groups, while all treatment were administered exclusively by the second investigator to minimize variability and bias. Anesthesia was administered using isoflurane (RWD, R510-22–10), with the trachea fully exposed in the anesthesia machine (Matrx, VIP 3000). The oxygen flow rate was maintained at 0.5–1 L/min, with an isoflurane concentration of 3% during induction and 1.5% during maintenance. Mice were euthanized by cervical dislocation 24 h after the final injection for sample collection. All procedures performed on animals were conducted in accordance with ARRIVE guidelines.

### In vivo tracking of CM-DiL-labeled hUC-MSCs

Cell tracking was performed using an IVIS Kinetic small animal live imaging system (PerkinElmer) at the Animal Center of Shantou University Medical College. Mice were anesthesia with isoflurane inhalation according to animal ethics protocols. Control and DN groups (n = 3/group) received injections of either PBS or CM-DiL-labeled hUC-MSCs (2 × 10^5^ cells/200μL PBS). Prior to injection, hUC-MSCs were incubated with 0.2 µM CM-DiL working solution (Bestbio, Shanghai, China) for 20 min at 37 °C followed by three PBS washes prior to transplantation. Then imaging was conducted at 8 h, 24 h, 48 h, 4 days, and 7 days post-injection.

### Histological analysis

Kidneys were sagittally sectioned, fixed in 10% neutral buffered formalin, paraffin-embedded, and cut into 4 μm sections. Tissue morphology was assessed by hematoxylin and eosin (HE; Wexis, China) and Masson trichrome (Kaiqi Biology, China) staining. Slides were imaged using an CX31 Olympus upright microscope (Olympus, Japan).

### Immunohistochemistry staining

Kidney sections were processed using the UltraSensitive SP Mouse/Rabbit Immunohistochemistry Kit (MXB, China). After blocking, sections were incubated overnight at 4 °C with primary antibodies: mouse anti-α-SMA (1:2000) and rabbit anti-TGF-β (1:1000), followed by species-matched secondary antibodies (goat anti-mouse/rabbit IgG). DAB development and hematoxylin counterstaining were performed after PBS washes. Sections were dehydrated through graded ethanol/xylene and imaged on an CX31 Olympus upright microscope (Olympus, Japan).

### Scanning electron microscope analysis

After the mice were euthanized via cervical dislocation, their kidney tissues were promptly excised within 1 min. The samples were then fixed in 2.5% glutaraldehyde solution and trimmed to around 0.5 mm^3^ before their skin was removed. Subsequently, the kidney tissue samples were washed thrice in PBS 15 min/wash. Following this, the tissue samples were infiltrated with osmium tetroxide for 1 h until a darkened appearance was achieved. After additional PBS washes (3 × 15 min), the tissue samples tissues were dehydrated through graded acetone series (30%, 50%, 70%, 90%, 100%; 30 min each) To prepare for the final viewing of the slices using the scanning electron microscope, a dehydration solution was meticulously prepared with specific ratios of anhydrous acetone and Embedding Agents A (1:3), B (1:5), and C (pure embedding agent) for a 12-h sequential tissue infiltration process. Ultrathin Sects. (70 nm) were collected on copper grids for SEM imaging (JEM-F200, Japan).

### Western blotting

Kidney tissues (around 20 mg each) were homogenized in 200 μL ice-cold strong RIPA lysis buffer (Solarbio, China) supplemented with 2μL PMSF, protease and phosphatase inhibitors. Protein concentrations were measured by BCA assay. Protein samples (around 10 μg) were loaded and separated on 10% SDS-PAGE gel, and transferred to polyvinylidene difluoride (PVDF) membrane (Millipore, US). After 1-h blocking with 5% non-fat milk, membranes were incubated overnight at 4 °C with primary antibodies: anti-Col3 (1:1000, ABclonal, China), anti-β-tublin (1:2000, CST, USA), and anti-α-SMA (1:1000, ABclonal, China). Following thrice washes with TBST, membranes were incubated with species-matched HRP-conjugated secondary antibodies (1:5000) for 1 h at room temperature. Specific protein bands were visualized using ECL reagent (NCM, China).

### RNA sequencing

Total RNA was extracted from hUC-MSCs using Trizol reagent (invitrogen USA). Libraries were sequenced on an Illumina HiSeq 6000 platform by Novogene. Differential gene expression analysis was performed with DEGSeq R package (version 1.16.1). Unsupervised hierarchical clustering of significantly dysregulated genes was visualized by heatmap. Pathway enrichment analysis utilized the KEGG database (*p* < 0.05).

### Statistical analysis

All data are presented as mean ± SEM and analyzed using GraphPad Prism 10. Statistical significance level was defined as *p* < 0.05. Group comparisons were performed by one-way or two-way ANOVA followed by LSD post-hoc tests where appropriate. Flow cytometry data were processed with modfit LT and FlowJo (BD Biosciences).

## Result

### The effects of hypoxia on biological characteristics of hUC-MSCs across serial passaging

To assess the impacts of oxygen concentrations on biological properties of hUC-MSCs during continuous passaging, in vitro differentiation assay revealed that hUC-MSCs cultured under both hypoxia and normoxia maintained adipocyte and osteoblast differentiation potential after serial passaging, although the capacity progressively diminished with increasing passage numbers (Supplemental Fig. 1A). Then flow cytometry analysis of surface marker expression showed that regardless of the oxygen concentration used during culture, high-passage hUC-MSCs consistently expressed CD29, CD44, CD73, CD90, and CD105, while remaining negative for HLA-DR, CD31 and CD34 (Supplemental Fig. 1B).

**Fig. 1:**
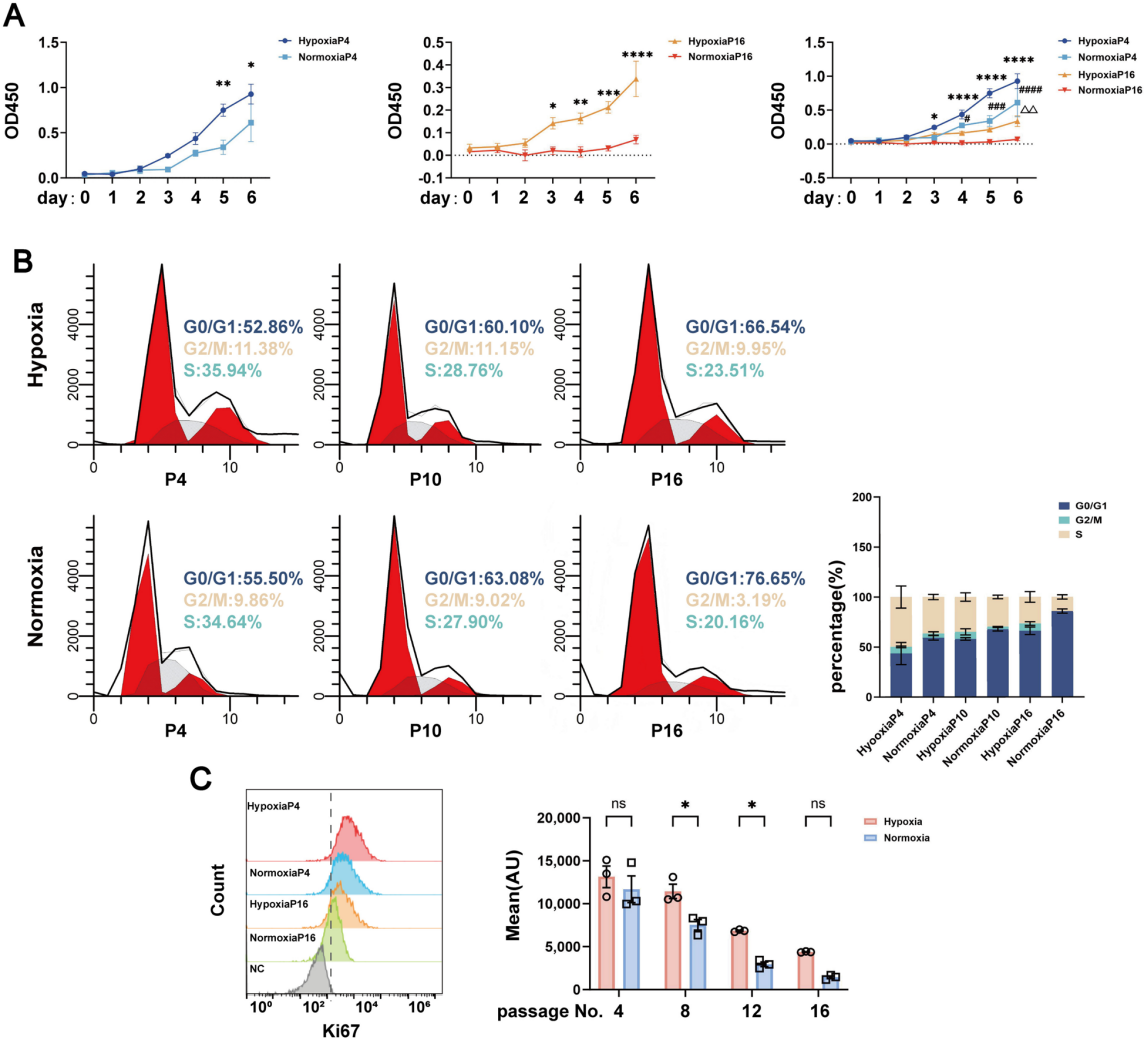
Effects of hypoxia on high-passage hUC-MSCs. (**A**) Proliferative capacity measured by CCK-8 assay (OD450) under normoxia (20%O_2_) and hypoxia (5%O_2_) at P4, P10, and P16 (days 0–6). PI staining. N = 3 (per group), data are mean ± SEM; *p < 0.05, **p < 0.01, ***p < 0.001, ****p < 0.0001 vs. corresponding Normoxia group; ^#^*p* < 0.05, ^###^*p* < 0.001, ^####^*p* < 0.0001 vs. corresponding Normoxia group; ^△△^*P* < 0.01 vs. corresponding Normoxia group. (**B**) Cell cycle distribution analyzed by (**C**) Ki67 staining mean fluorescence intensity (MFI) quantification measured by Flow cytometry. N = 3 per group; ns = no significance, *p < 0.05 vs. corresponding Normoxia group

### Hypoxia increases and maintains cell proliferative capacity of hUC-MSCs during serial passaging

To assess the impact of oxygen levels on cell proliferation, the CCK-8 assay revealed significantly enhanced proliferation cultured under 5%O_2_ hypoxia versus 20%O_2_ normoxia across serial passages(P4-P16), evidenced by elevated OD450 values over 7-day culture. Strikingly, high-passage(P16) normoxic cultured cells exhibited near-complete proliferative arrest, while hypoxic cultured Passage16 hUC-MSCs retained relatively high proliferative capacity (Fig. 1A).

To further confirm this phenomenon, the expression of cell proliferation marker, Ki67 and cell cycle were also determined by flow cytometry after Immunofluorescence staining and PI staining. In consistent with CCK-8 assay, cell cycle profiling revealed that normoxia induced significant G0/G1 phase accumulation and decline in the S + G2/M phase populations, while hypoxia suppressed G0/G1 arrest and high-passage(P16) cells maintained significantly lower G0/G1 proportions than normoxic counterparts (Fig. 1B). The Ki67 staining results showed that hypoxic cultures maintained markedly higher fluorescence intensity versus normoxia, indicating sustained proliferative potential. However, progressively diminished mean fluorescence intensity in normoxia was observed with continuous passages (Fig. 1C). Collectively, although in vitro serial passaging progressively impairs hUC-MSCs proliferation regardless of oxygen levels, physiologically-relevant hypoxia (5%O_2_) dramatically mitigates this decline, preserving > 30% cycling cells even at late passages(P16) through reduced G0/G1 blockade and sustained proliferative marker expression.

### Hypoxic culture conditions protects hUC-MSCs from oxidative damage and effectively delays the cellular senescence during extended serial passaging

**Fig. 2:**
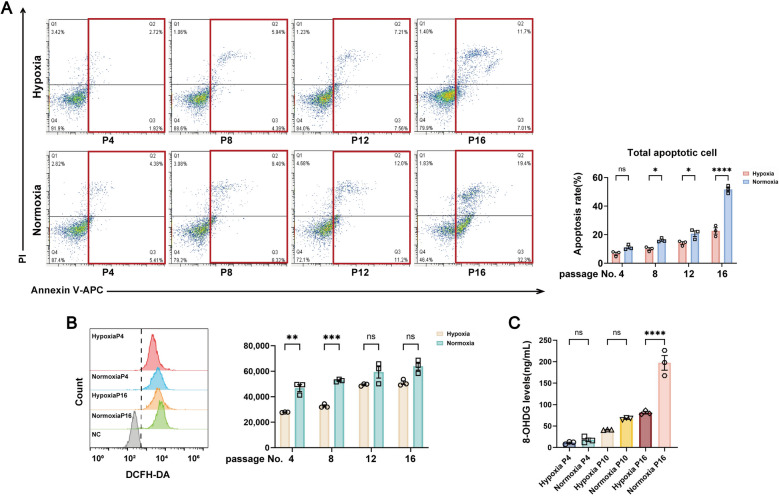
Hypoxia attenuates apoptosis and oxidative stress in high-passage hUC-MSCs. (**A**) Flow cytometry plots of Annexin V-APC/PI staining under normoxia (20%O_2_) and hypoxia (5%O_2_) at P4, P8, P12, and P16. Quadrants: Q1 (necrotic), Q2 (late apoptotic), Q3 (viable), Q4 (early apoptotic). Quantification of total apoptotic cells (Q2 + Q3). (**B**) The ROS levels measured by DCFH-DA mean fluorescence intensity (MFI). (**C**) 8-OHDG concentrations in the supernatant (DNA oxidative damage marker). N = 3 (per group); data are mean ± SEM. Significance vs. Normoxia group: ns = no significance, *p < 0.05, **p < 0.01, ***p < 0.001, ****p < 0.0001

To ascertain the impact of continuous passaging and oxygen tension on cellular integrity of hUC-MSCs, Annexin V/PI flow cytometry analysis revealed a significant, oxygen level-dependent elevation in apoptosis rates during serial passaging (P4-P16). Normoxic cultured high-passage(P16) cells exhibited profound apoptosis rate, whereas hypoxic cultures markedly attenuated both early and late apoptosis (Fig. 2A). Since 20%O_2_ is sharply higher than the physiological concentration, it may cause oxidative stress and DNA damage [16]. We measured the levels of ROS and 8-OH guanine, a DNA oxidative damage marker. Our results showed that the ROS levels were higher in hUC-MSCs cultured under 20%O_2_ normoxia versus 5%O_2_ hypoxia and kept increasing during serial passaging (Fig. 2B). Accordingly, hypoxia substantially reduced 8-OH guanine level, particularly in high-passage(P16) cells compared with normoxia (hypoxia: 80.99 ± 2.83 ng/mL vs. normoxia 197.20 ± 17.02), thereby indicating the protective role of hypoxia against oxidative damage (Fig. 2C).

Given that oxidative stress leads to damage of biomolecules such as DNA, proteins, and lipids, thereby contributing to cellular senescence, we next explored the impact of oxygen levels on cell senescence during serial passaging by detecting different hallmarks. The morphological alteration like enlarged and fattened cell body is the key feature of in vitro senescence, flow cytometric analysis of cell size showed that hUC-MSC volumes increased during passaging (P4-P18), as evidenced by a rightward shift on the forward scatter (FSC, indicating the cell size) axis [27]. Notably, 5%O_2_ hypoxic conditions markedly suppressed cell size increase in high-passage(P12-P18) hUC-MSCs compared with 20%O_2_ normoxia (Supplemental Fig. 2A).

**Fig. 3:**
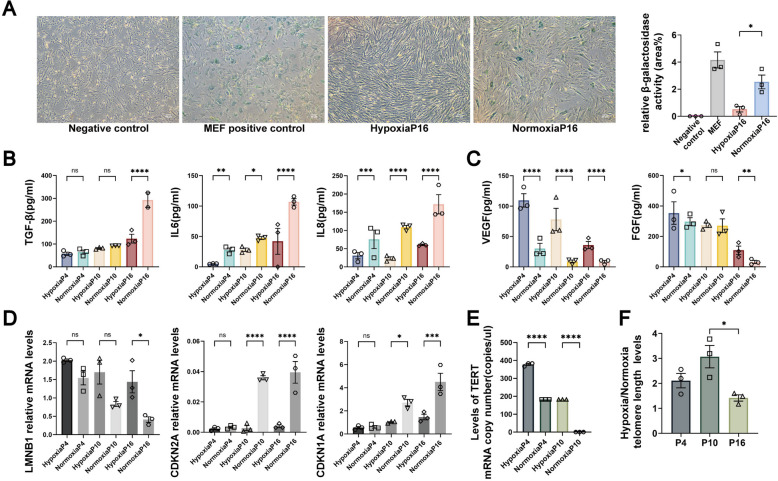
Hypoxia mitigates senescence phenotypes in hUC-MSCs. (**A**) SA-β-gal staining (green-yellow: senescent cells). quantitative analysis of SA-β-gal positive area (normalized to total cell area, area%). Groups: Negative control (Neg), MEF, mouse embryonic fibroblasts (positive control). (**B**, **C**) Secretome analysis by ELISA: TGF-β, IL-6, IL-8, and VEGF, FGF concentrations in the supernatant. (**D**, **E**) mRNA expression of senescence markers (*LMNB1*, *CDKN2A*, *CDKN1A*) and telomerase (*hTERT*) by qRT-PCR. (F) Relative telomere length quantification. Data = mean ± SEM (n = 3). *p < 0.05, **p < 0.01, ***p < 0.001, ****p < 0.0001 vs. Normoxia P16 group. ns = no significance. Scale bar indicates 50 μm

Then, we detected senescence-associated β-galactosidase activity, a widely used marker for cellular aging. β-Galactosidase staining revealed that normoxic (20%O_2_) cultures (P16) accumulated blue-green senescent cells markedly earlier and more than their hypoxic (5%O_2_) counterparts, confirming the anti-aging advantage of low-oxygen expansion (Fig. 3A). Mouse embryonic fibroblasts (MEF) were used as a positive control for SA-β-gal staining.

Senescence-Associated Secretory Phenotype (SASP) refers to the complicated and dynamic secretory profile of senescent cells, including a wide range of chemokines, cytokines, growth factors, and proteases. ELISA analysis revealed that the levels of SASP factorssuch as IL-6, IL-8, and TGF-β kept increasing in supernatants of hUC-MSCs cultured under 20%O_2_ during serial passaging particularly in passage16, whereas hypoxia dramatically inhibited the secretion of these factors in high-passage hUC-MSCs (Fig. 3B). In contrast, hypoxia maintained growth factor secretion, including VEGF and FGF in high-passage hUC-MSCs compared to normoxic culture condition (Fig. 3C).

We next determined the expression of biomarkers of senescence including *CDKN2A*, *CDKN1A* and *LMNB1*. RT-qPCR analysis showed that hypoxia was associated with reduced expression of *CDKN2A* and *CDKN1A*, and preservation of *LMNB1* expression across serial passaging compared to normoxic conditions (Fig. 3D).

Given the established link between replicative senescence and telomere attrition, we further measured telomerase expression and telomere length. Quantitative PCR analysis revealed significant upregulation of telomerase catalytic subunit *hTERT* (182.44 copy versus 0 copy) in hypoxic P10 hUC-MSCs compared to normoxic controls (Fig. 3E). Consistent with enhanced telomerase activity, telomere length was maintained at 3.19-fold higher levels under hypoxia in P10 cells and substantially preserved telomere length till P16 (1.17 folds) (Fig. 3F). These results demonstrate that hypoxia mitigates replicative senescence by preserving telomere integrity partially through *hTERT*-mediated mechanisms. These findings collectively demonstrate that 5%O_2_ culture conditions are more effective in inhibiting cell size enlargement, suppressing the production of SASP factors, and delaying the processes of cellular growth and aging.

### RNA sequencing (RNA-Seq) reveals hypoxia-delayed senescence through suppression of pro-inflammatory pathways

To elucidate the molecular mechanisms underlying the anti-senescence effects of hypoxic culture conditions on hUC-MSCs, RNA-Seq analysis was performed on low-passage and high-passage hUC-MSCs cultured under both 5%O_2_ hypoxic and 20%O_2_ normoxic conditions.

**Fig. 4:**
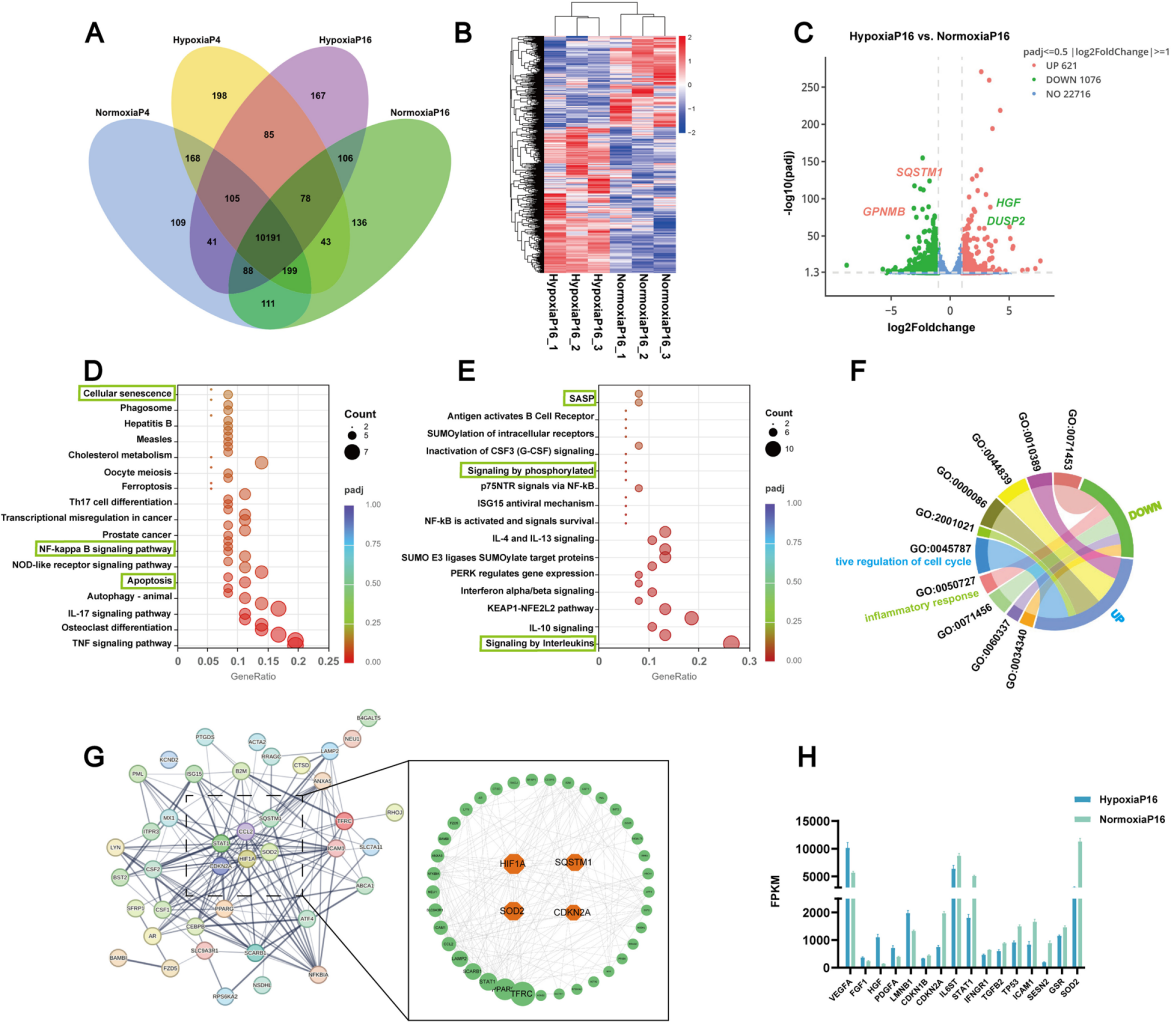
Transcriptomic profiling reveals hypoxia-regulated pathways in hUC-MSCs. (**A**) Venn diagram of overlapping gene expression between hypoxia (5%O_2_) and normoxia (20%O_2_) groups. (**B**) Heatmap of differentially expressed genes (DEGs) gene expression patterns. (**C**) Volcano plot of DEGs. (**D**) KEGG pathway analysis. (**E**) Reactome pathway analysis. (**F**) Gene Ontology (GO) enrichment analysis. (**G**) Protein–protein interaction (PPI) analysis. (**H**) Validation of key DEGs by FPKM value of genes. N = 3 (per group, biological replicates), data = mean ± SEM

Comparative transcriptomic profiling of four experimental groups, low-passage(P4) and high-passage(P16) hUC-MSCs under hypoxia (5%O_2_) versus normoxia (20%O_2_) identified 11,825 differentially expressed genes (DEGs) as illustrated in Fig. 4A. Hierarchical clustering of DEGs revealed distinct transcriptional profiles between the groups. Notably, compared to high-passage(P16) hUC-MSCs cultured under 20%O_2_ normoxic conditions, the expression of genes such as pro-aging mediators *GPNMB* and *SQSTM1* were significantly downregulated, while rejuvenation-associated regulators like *DUSP2* and *HGF* were significantly upregulated in hypoxic P16 cells (Fig. 4B, C). To validate the hypothesis that hypoxic culturing mitigates replicative senescence by modulating cell-cycle progression and suppressing the SASP, KEGG, Reactome, and GO enrichment analyses of the DEGs identified significant enrichment in pathways governing cell-cycle regulation, cellular senescence, and growth factor secretion (Fig. 4D–F). The protein–protein interaction (PPI) network further confirmed significant downregulation of proteins related to senescence-driving pathways, including downregulation of the cell cycle inhibitor CDKN2A, the NF-κB pathway activator SQSTM1, and the antioxidant-related protein SOD2. In contrast, cultivation under normal oxygen conditions promotes cellular senescence by inhibiting the cell cycle and upregulating oxidative stress pathways (Fig. 4G). FPKM quantification revealed significant suppression of pro-inflammatory cytokines such as *IL-6*, *IFN*, and *TGF-β* coupled with enhanced expression of *VEGF* and *FGF,* growth factors in hypoxic high-passage hUC-MSCs (Fig. 4H). Hypoxia delays senescence through transcriptional inhibition of pro-inflammatory and pro-aging pathways while promoting cell-cycle progression and regenerative factor secretion.

### Establishment of diabetic nephropathy mouse model and homing capacity of hUC-MSCs to damaged renal tissue

**Fig. 5:**
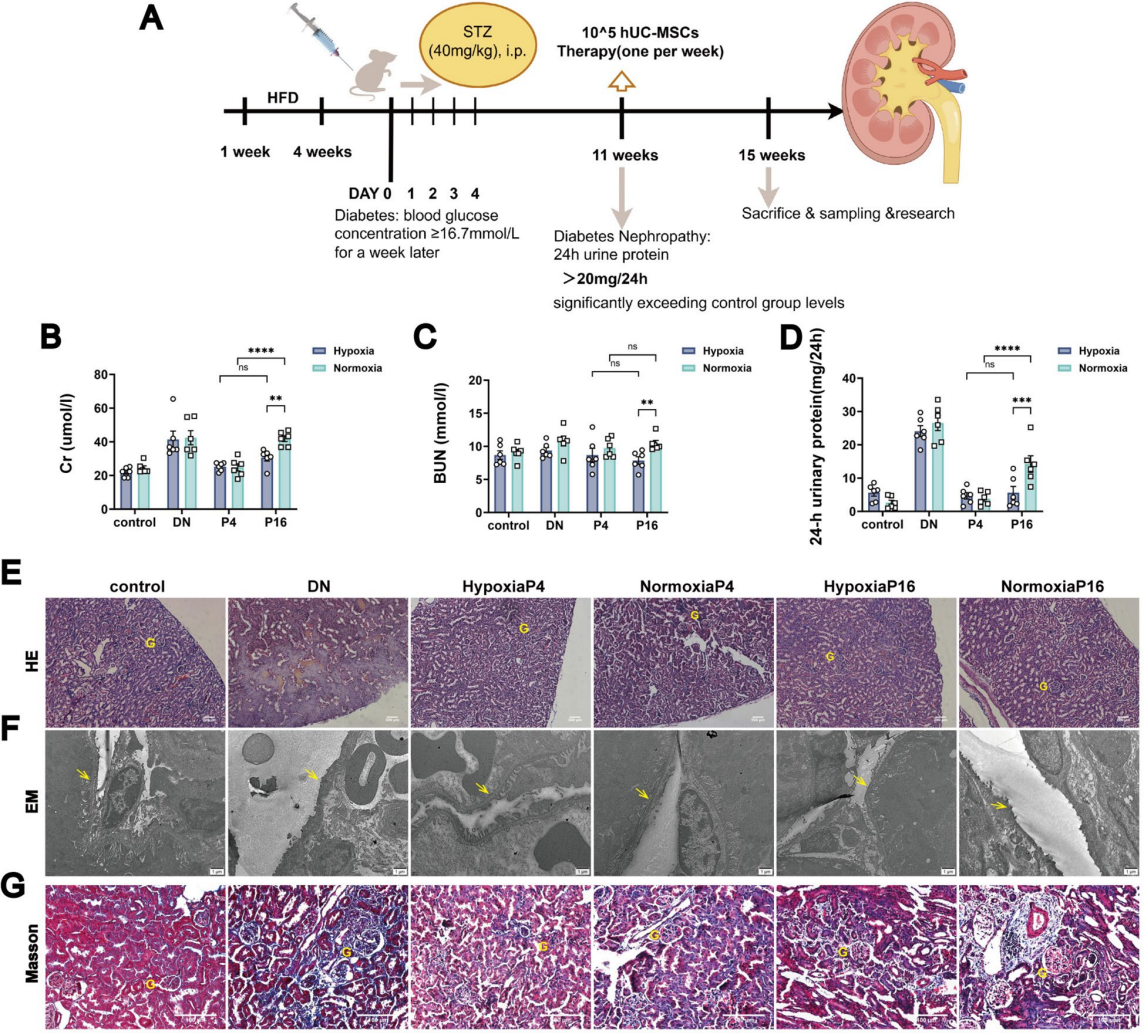
Hypoxic high-passage hUC-MSCs ameliorate renal injury in DN mice. (**A**) Experimental design and timeline of DN model establishment by Figdraw. (**B**-**D**) Level changes of renal function markers after transplantation of hUC-MSCs: (B)Serum creatinine (Cr), (**C**) Blood urea nitrogen (BUN), (**D**) 24-h urinary protein. The transplantation of high-passage hUC-MSCs cultured under 5%O_2_ hypoxia resulted in better preservation of kidney injury markers. (**E**) H&E staining of kidney tissue. (**F**) Electron microscopy examination of kidney tissue. (**G**) Masson trichrome staining of kidney tissue quantifying renal fibrosis (blue: collagen deposition). Groups: Control (healthy), DN (untreated), Hypoxia P4/P16 (5%O_2_-cultured hUC-MSCs), Normoxia P4/P16 (20%O_2_-cultured hUC-MSCs). N = 4 (per group), data = mean ± SEM. *p < 0.05, ***p < 0.001 *vs.* Normoxia P16 group. G:glomerulus. Scale bars: 200 μm (E), 1 μm (F), 100 μm (G)

To obtain the in vivo evidence of the therapeutic potential of high-passage hUC-MSCs cultured under hypoxia, a type 2 diabetic nephropathy mouse model was established via HFD and STZ injection. Following STZ treatment, the DN group exhibited random blood glucose levels exceeding 16.7 mmol/L from the 11th week indicating successful establishment of the diabetic model. Then, followed by a 4-week period of tail vein injection of hUC-MSCs (1 × 10^5^ cells/week), the animals were euthanized, and samples were collected for further analysis (Fig. 5A). Notably, progressive body weight loss and was observed in DN mice post-disease induction, with a reduction from 35.18 g to 29.05 g at week 15, lower than control group(34.05 g), along with elevated blood glucose levels (Supplemental Fig. 3A, B). Furthermore, levels of Cr, BUN, and 24-h urine protein in DN mice significantly increased at week 11 and week 15 compared to the control group, with urine protein exceeding 20 mg/24 h at the 11th week (Supplemental Fig. 3C–E). In addition, urine protein levels exceeded 20 mg/24 h at week 11, significantly higher than threefold that of the control group. Strikingly, administration of hUC-MSCs(P6, cultured under normoxia) at week 11 effectively attenuated these elevations (Supplemental Fig. 3C–E).

To track in vivo biodistribution, CM-Dil-labeled hUC-MSCs were intravenously infused into DN mice and homing dynamics were monitored via in vivo small animal imaging at different time points within one week (Supplemental Fig. 4A). Systemic biodistribution analysis revealed peak fluorescence intensity at 24 h post-infusion and then gradually decreased (Supplemental Fig. 4B). Intriguingly, DN kidneys exhibited higher CM-Dil signal intensity versus non-diabetic controls (11.73 ± 0.37 vs. 9.81 ± 0.80 radiance units), confirming renal-targeted homing of hUC-MSCs to injured kidney (Supplemental Fig. 4C).

### Hypoxic culture preserves therapeutic efficacy of high-passage hUC-MSCs in diabetic nephropathy mice: Improved renal function and pathological injury

As our data found the protective roles of hypoxia against cellular senescence in vitro, we further investigated whether hypoxia was able to maintain the therapeutic potential of hUC-MSCs across serial passaging using diabetic nephropathy mouse model. Four group of cells, low-passage(P6) and high-passage(P16) hUC-MSCs cultured under hypoxia (5%O_2_) and normoxia (20%O_2_), were administered via tail vein injection from week 11 as shown in Fig. 5A, once per week for four weeks. The blood tests results showed that transplantation of both hypoxic and normoxic low-passage(P6) hUC-MSCs significantly ameliorated renal dysfunction in DN mice compared to control group, no difference of therapeutic efficacy was found between these two types of cell. In contrast, the therapeutic efficacy of high-passage(P16) hUC-MSCs varied depending on O_2_ tension. Administration of hypoxic P16 hUC-MSCs markedly decreased the levels of Cr, BUN, and 24-h urine protein in DN mice. MSC-treated DN mice exhibited 34.63% lower Cr (28.80 ± 2.60 vs. 44.06 ± 7.37 μmol/L), 45.12% reduced BUN (7.23 ± 0.60 vs. 13.18 ± 1.11 mmol/L), and 81.57% decreased 24-h proteinuria (4.61 ± 1.79 vs. 25.02 ± 1.19 mg/24 h) compared to untreated DN controls, while normoxic P16 hUC-MSCs treatment showed no therapeutic effect (Fig. 5B–D).

To compare and evaluate how the oxygen milieu during passaging influences the reparative potency of hUC-MSCs, we subjected murine kidneys to histological and ultrastructural interrogation. HE and Masson’s trichrome staining revealed extensive inflammatory-cell infiltration, glomerulor enlargement, hyaline arteriolar change, exuberant collagen deposition within glomeruli and the tubulointerstitium of DN kidneys (Fig. 5E, G). These lesions were markedly reduced after treatment with low-passage(P4) and hypoxic high-passage(P16) hUC-MSCs, whereas infusion of normoxic high-passage(P16) hUC-MSCs did not show much improvement.

We next investigate and compare the tissue repair capacity of different hUC-MSCs at the ultrastructural level, TEM disclosed pronounced thickening of the glomerular basement membrane (GBM) and widespread effacement of podocyte foot processes in DN kidneys. Mice receiving hypoxic high-passage hUC-MSCs exhibited near-normal GBM thickness and restoration of a well-defined interdigitating foot-process architecture. Conversely, DN kidneys treated with 20%O_2_-expanded high-passage hUC-MSCs still displayed segmental GBM redundancy and incomplete podocyte repair or protection (Fig. 5F). These data establish that hypoxic culture conditions preserve superior anti-inflammatory, anti-fibrotic, and glomerular-protective properties of high-passage hUC-MSCs, enhancing their therapeutic potential in DN. In summary, the therapeutic efficacy of these cells showed significant oxygen-dependence and passage-dependency during expansion.

### Hypoxia sustains potency of high-passage hUC-MSCs potentiating anti-fibrotic mechanisms in DN kidneys

**Fig. 6:**
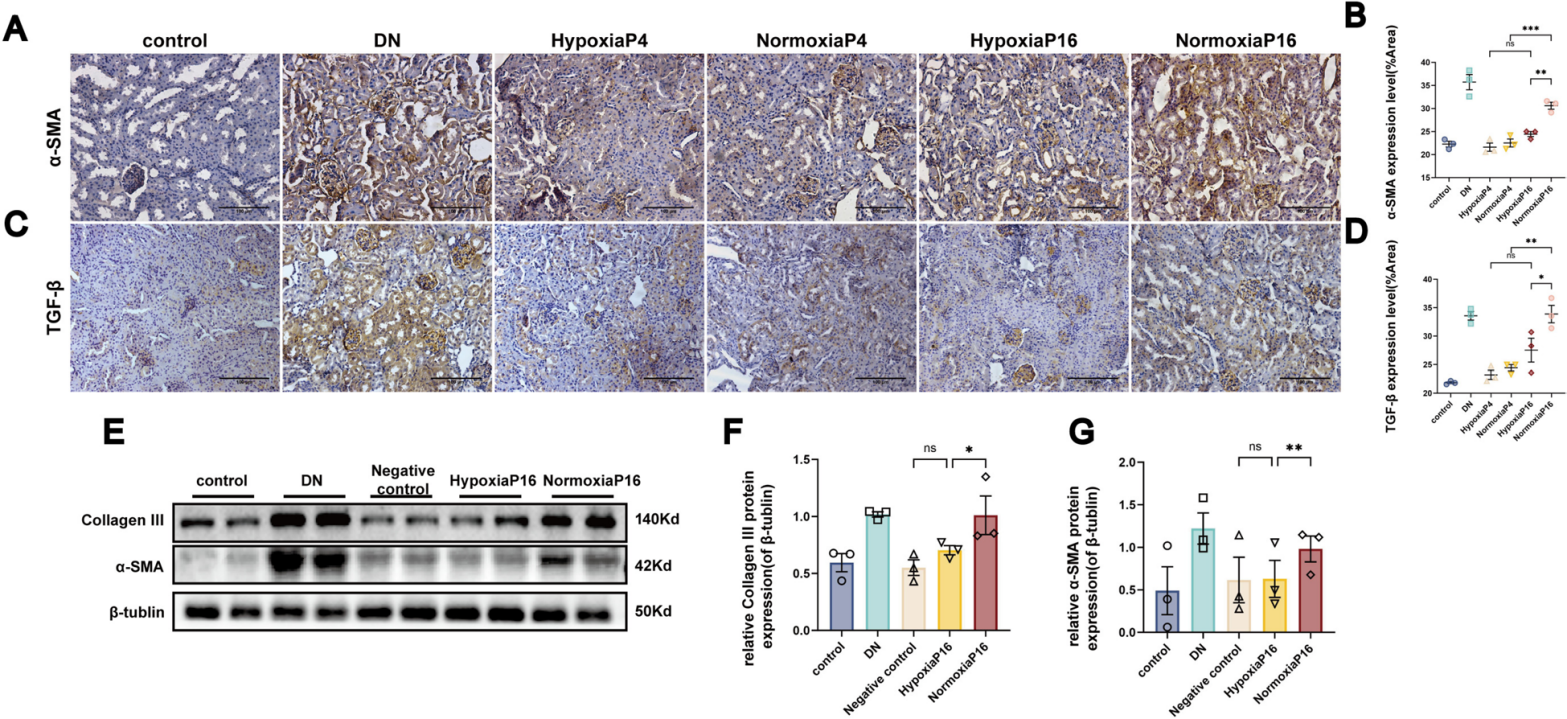
Hypoxic high-passage hUC-MSCs attenuate renal fibrosis in DN. (**A**) Immunohistochemical staining (IHC) of α-SMA in kidney tissues (groups: Control, DN, HypoxiaP4, NormoxiaP4, HypoxiaP16, NormoxiaP16). (**B**) Quantification of α-SMA positive area (%Area). (**C**) IHC of TGF-β in kidney tissues (same groups as A). (**D**) Quantification of TGF-β positive area (%Area). (**E**) Western blot analysis of fibrosis markers: Collagen III and α-SMA protein. (**F**) Quantification of Collagen III protein expression (normalized to β-tublin). (**G**) Quantification of α-SMA protein expression (normalized to β-tublin). N = 4 (per group), data = mean ± SEM. *p < 0.05, **p < 0.01 *vs*. NormoxiaP16 group. Groups: Control (healthy), DN (untreated diabetic nephropathy), Hypoxia P4/P16 (DN + 5%O_2_-cultured hUC-MSCs), Normoxia P4/P16 (DN + 20%O_2_-cultured hUC-MSCs). Scale bars: 100 μm (A)

Since our Masson’s staining found hUC-MSCs exuberant collagen deposition within glomeruli and the tubulointerstitium of DN kidneys, to dissect how the culture oxygen tension influences the anti-fibrotic capacity of serial passaged hUC-MSCs, we further profiled key fibrogenic markers. Immunohistochemistry revealed robust up-regulation of α-SMA and TGF-β in DN kidneys (Fig. 6A–D). High-passage hUC-MSCs expanded at 5%O_2_ markedly attenuated this expression, whereas cells cultured at 20%O_2_ conferred only modest suppression. Western blot confirmed these findings: treatment with 5%O_2_-expanded MSCs significantly reduced renal Collagen III and α-SMA protein levels relative to their 20%O_2_ counterparts (Fig. 6E–G). Thus, hypoxia preserves the intrinsic anti-fibrotic efficacy of high-passage hUC-MSCs, limiting myofibroblast activation and extracellular-matrix deposition in diabetic kidneys.

### RNA-Seq reveals hypoxia-preserved renoprotective capacity of high-passage hUC-MSCs via transcriptional repression of inflammatory and fibrosis pathways

**Fig. 7:**
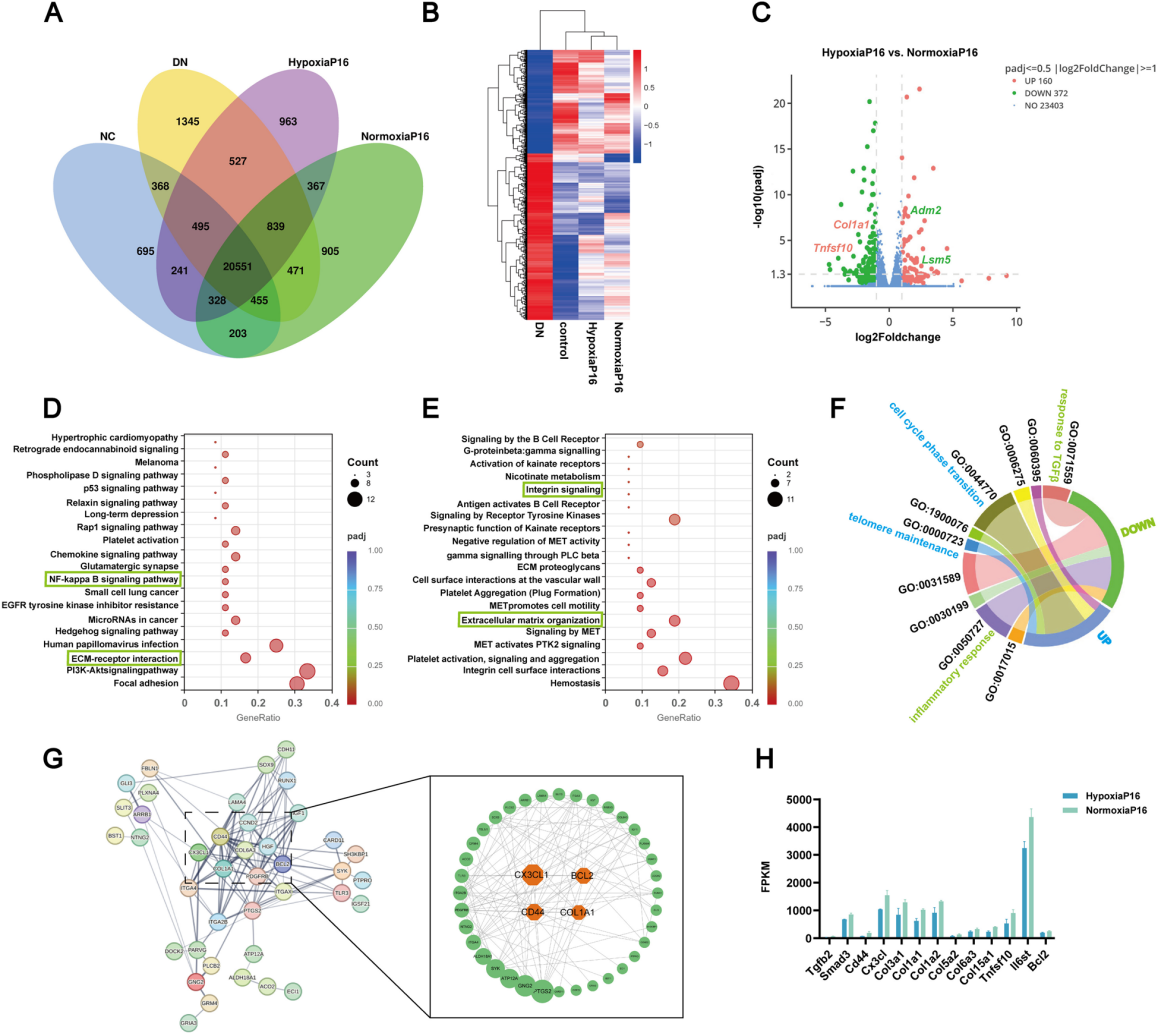
Transcriptomic profiling of renal tissues reveals potential molecular mechanisms underlying MSC therapy efficacy. (**A**) Venn diagram of overlapping gene expression between different groups: NC group (healthy control), DN group (untreated), Hypoxia group (DN + 5%O_2_-cultured hUC-MSCs), Normoxia group (DN + 20%O_2_-cultured hUC-MSCs). (**B**) Heatmap of differentially expressed genes (DEGs) gene expression patterns. (**C**) Volcano plot of DEGs. (**D**) KEGG pathway analysis. (**E**) Reactome pathway analysis. (**F**) Gene Ontology (GO) enrichment analysis. (**G**) Protein–protein interaction (PPI) analysis. (**H**) Validation of key DEGs by FPKM value of genes. N = 3 (per group, biological replicates), data = mean ± SEM

To dissect how oxygen tension during expansion influences the in vivo tissue repair capacity and possible mechanisms of high-passage hUC-MSCs, we profiled whole-kidney transcriptomes after four-week transplantation of high-passage(P16) hUC-MSCs cultured under 5% or 20%O_2_. RNA-Seq of whole-kidney tissue identified 28,753 differentially expressed genes (DEGs) between hypoxic and normoxic hUC-MSCs recipients (fpkm > 1) (Fig. 7A). Hierarchical clustering highlighted distinct transcriptomic signatures in hypoxic P16 hUC-MSCs recipients diverged sharply from their normoxic counterparts: significant downregulation of collagen proliferation and inflammatory fibrosis genes like *Col1a1* and *Tnfsf10*, whereas selective upregulation of cell- homeostasis related genes *Adm2* and *Lsm5*, indicating a metabolic shift from proliferative to homeostatic programs (Fig. 7B, C). KEGG, Reactome, and GO functional enrichment analyses converged on NF-κB signaling pathway, ECM-receptor interaction, and Extracellular matrix organization as the most prominently enriched pathways (Fig. 7D–F). The PPI network further indicated that P16 kidneys cultured under hypoxic conditions exhibited significant suppression of the inflammatory response pathway NF-κB, along with marked inhibition of classical pro-fibrotic responses, including downregulation of cell adhesion proteins Cx3cl1 and Cd44, excessive extracellular matrix deposition proteins Col1a1 and Col6a3, pro-fibrotic TGF-β signaling pathway-associated proteins Itgav and Fbln1, as well as NF-κB pathway-related Bcl2 proteins. Although residual transcripts for *Tgfb* and *Col3a1* remained detectable, their abundance was significantly lower than in kidneys treated with normoxia-expanded hUC-MSCs (Fig. 7G, H). Our data indicate that hypoxia preserves the renoprotective potency of high-passage hUC-MSCs by transcriptionally repressing fibrosis- and inflammation- associated gene networks while reinforcing metabolic and structural homeostasis within the injured diabetic kidney.

## Discussion

Mesenchymal stem cells derived from various sources hold transformative potential in the field of regenerative medicine, tissue repair, and basic therapeutic strategies due to their unique biological properties [28]. As we mentioned above, In vitro expansion of MSCs is the critically essential prerequisite for their clinical application, and the quality of cells is the key determinant of therapeutic effectiveness. It is therefore of great significance optimizing expansion protocols for MSCs to ensure the cell quality and quantity. Oxygen is an essential nutrient and plays a crucial role in cellular aerobic metabolism, particularly the tricarboxylic acid cycle [29]. Thus, oxygen tension is also a vital environmental parameter in cell culture and an important consideration for optimizing cell culture protocols, directly influencing cell viability and function.

Previous studies have largely highlighted the beneficial effects of physiological hypoxic culture conditions (1–5%O_2_) over conventional atmospheric oxygen levels (20%O_2_) on MSCs [30–33]. Yet, rigorous data elucidating the impact of hypoxia on MSC senescence and biological activity during long-term serial passaging remain limited, particularly in vivo evidence is still absent. Here, we systemically evaluated and compared the impacts of different oxygen tensions on hUC-MSC cellular senescence and functions during serial passaging, both in vitro and in vivo. Our data confirm that culturing under hypoxic condition (5%O_2_) effectively delays replicative senescence and maintains the bioactivity of hUC-MSCs during serial passaging in vitro. Importantly, for the first time, we provided the in vivo data supporting that hypoxic culture conditions preserve the therapeutic efficacy of hUC-MSCs after continuous passaging till high passage numbers making use of DN mouse model.

In 1961, Hayflick and Moorhead reported limited proliferative capacity of primary human fibroblasts in vitro which was the first formal description of cellular senescence [34]. Following studies demonstrate that even stem cells including MSCs also undergoes cellular senescence during in vitro expansion in spite of their self-renewal capacity. Exogenous and endogenous factors that induce cell damage or stress—including DNA damage, dysfunctional telomeres, and genome instability—contribute to cellular senescence. Oxidative stress from environmental oxygen levels is one of the key contributing factors. Our results showed that ROS levels were higher under 5%O_2_ hypoxia than 20%O_2_ normoxia and increased with serial passaging. However, hypoxia significantly reduced oxidative DNA damage, especially in high-passage(P16) cells. Consequently, the detailed detection of cell senescence hallmarks such as cell enlargement [35], β-galactosidase activity [36], secretion of SASP [37, 38], and biomarkers of senescence (*CDKN2A*, *CDKN1A* and *LMNB1*) [39] confirmed that hypoxia effectively delays replicative senescence of hUC-MSCs during in vitro serial passaging.

Given that cellular senescence entails permanent growth arrest, we further examined cell cycle progression and proliferation in our model. During continuous passaging of hUC-MSCs under either 5%O_2_ or 20%O_2_ concentrations, a key finding was that hypoxic culture significantly enhanced cell proliferation across both low- and high-passage cells compared to normoxia, while a marked decline in proliferative capacity was observed predominantly in normoxic hUC-MSCs during passaging. Accordingly, hypoxic high-passage hUC-MSCs also exhibited a marked reduced G0/G1-phase cell cycle arrest and significantly lower apoptotic rate relative to their normoxic counterparts. Most previous studies reported similar findings, but only a few documented inhibition of cell growth in hypoxia [40].

Telomeres are highly conserved short tandem repeats located at the end of chromosomes in eukaryotic cells and play key roles maintaining genomic stability and integrity. Any factor causing telomere attrition contributes to senescence and telomere shortening also become one of the features of cell senescence [41].

Telomeric DNA is synthesized by telomerase, a specialized reverse transcriptase, but not replicative DNA polymerases [42]. Accordingly, most somatic cells and stem cells with too low level or without telomerase expression will undergo gradual telomere shortening after several rounds of cell division, which induces genomic instability and further leads to cell death or replicative senescence [43].

Consistent with our observation that 5%O_2_ hypoxic culture effectively delays replicative senescence in serially passaged hUC-MSCs, we also found that hypoxia significantly attenuated telomere shortening compared to normoxia, even at high passage numbers. Similar results were reported by Estrada et al. using 3%O_2_[44]. This effect may be attributed to the elevated *hTERT* expression level (Fig. 3E) under hypoxic condition, which could partially compensate for telomere attrition during continuous passaging—despite the fact that *hTERT* mRNA levels remain relatively low and even have previously been reported as undetectable in MSCs [45]. Mechanistically, the hypoxia-induced upregulation of *hTERT* may be mediated by HIF-1α, which has been reported to directly transactivate the *hTERT* promoter via binding to two hypoxia response elements located in its proximal promoter region [46]. Such HIF-1α-mediated transcriptional regulation provides a plausible mechanistic explanation for our observation that hypoxic culture increases *hTERT* mRNA expression in hUC-MSCs. Of note, the regulation of *hTERT* by hypoxia appears to be multilayered. In addition to such transcriptional activation, hypoxia has also been reported to promote alternative splicing of *hTERT* pre-mRNA toward the active variant [47]. Such post-transcriptional regulation may further contribute to the increased *hTERT* mRNA expression observed under hypoxic conditions.

Additionally, RNA sequencing analysis of low-passage(P4) and high-passage(P16) hUC-MSCs cultured under 5%O_2_ and 20%O_2_ respectively, further supported our findings. In high-passage hUC-MSCs maintained under 5%O_2_, mRNA expression of pro-inflammatory factors such as *IL-6*, *IFN*, and *TGF-β* was reduced, while the mRNA expression of growth factors associated with anti-inflammatory and anti-fibrotic effects (VEGF, FGF, HGF, PDGF) was significantly increased. These findings align with previous reports confirming the roles of these factors in anti-fibrotic and anti-inflammatory processes [31, 48, 49]. Moreover, hypoxic high-passage(P16) hUC-MSCs exhibited downregulation of genes associated with oxidative stress (*TP53*,*ICAM1*), followed by a decrease in the stimulation of antioxidant genes (*SESN2*,GSR,*SOD2*), underscoring a synergistic interaction between oxidative stress and inflammatory responses [50–54]. Mechanistically, it is well established that ROS, the key mediators of oxidative stress, can stimulate the production of pro-inflammatory cytokines including TNF-α and IL-6, which further amplify ROS production, thereby forming a self-reinforcing vicious cycle that sustains chronic inflammation [55, 56]. Together, our results demonstrate that hypoxia effectively interrupts this positive feedback loop between oxidative stress and inflammatory responses.

Then we hypothesized that the 5%O_2_ hypoxic environment may suppress the production of aging-associated secretory factors via a paracrine mechanism, thereby reducing oxidative stress-induced damage and delaying the emergence of cellular senescence phenotypes, while preserving cellular repair activity. Gene Set Enrichment Analysis (KEGG, Reactome) on differentially expressed genes from RNA sequencing revealed that hypoxic high-passage hUC-MSCs downregulated multiple pathways promoting inflammatory responses, including NF-κB, interleukin, SASP signaling and TGF-β signaling. Concurrently, pathways related to oxidative phosphorylation, the cell cycle, and cellular aging were also suppressed. These pathways interact and exhibit positive regulatory effects on each other. Previous studies indicate that they regulate the expression of pro-inflammatory factors and play key roles in mediating inflammatory responses [57, 58]. Among these interconnected signaling pathways, the crosstalk between TGF-β and NF-κB has been well documented. Accumulating evidence indicates that TGF-β interacts with NF-κB to synergistically drive renal inflammation and fibrotic progression [59]. The concurrent downregulation of both pathways under hypoxia therefore suggests that low oxygen tension attenuates this synergistic crosstalk, which is consistent with our observation of reduced inflammatory and fibrotic gene expression in hypoxic hUC-MSCs.

Thus far, we have systemically evaluated and compared the effects of hypoxic vs normoxic conditions on hUC-MSCs during prolonged in vitro passaging. In agreement with most previous reports, our results indicate that hypoxic culture effectively delays cellular senescence and preserves biological functions such as growth factor secretion of MSCs. However, all these findings are obtained from in vitro models, and the robust in vivo evidence supporting the maintenance of therapeutic efficacy in hypoxic-cultured MSCs after extended passaging remains lacking. To address this gap, we performed in vivo transplantation experiments using a mouse model of type 2 diabetic nephropathy. DN is a severe and progressive complication of diabetes with limited therapeutic options, representing one of the leading causes of end-stage renal disease and mortality [60]. The STZ combined with HFD model was selected based on the following rationale: STZ induces pancreatic β-cell apoptosis and consequent hyperglycemia, whereas HFD promotes insulin resistance and metabolic disorders. As summarized in recent reviews, this combined strategy reliably recapitulates the metabolic features of human type 2 diabetes mellitus [61], and has been widely validated for establishing stable diabetic kidney disease models in mice with the capacity to mimic key pathological features including albuminuria, glomerulosclerosis, and tubulointerstitial fibrosis [62]. Regarding MSC-based therapy for DN, most previous studies have focused on bone marrow-derived MSCs (BM-MSCs) or adipose-derived MSCs (AD-MSCs), which have been shown to improve glycemic control, ameliorate proteinuria, and suppress renal inflammation [63]. However, the therapeutic potential of hUC-MSCs, particularly those cultured under different oxygen tensions during in vitro expansion, remains less explored in the context of DN. Based on our in vitro findings that hypoxic culture preserves the anti-senescent and paracrine functions of hUC-MSCs, we further investigated whether these beneficial properties could translate into enhanced reno-protective efficacy in vivo using the STZ/HFD-induced DN model.

We first detected the biodistribution of CM-Dil-labeled hUC-MSCs by in vivo small animal imaging after intravenous infusion, and our results (Fig. S4B) revealed fluorescent signals primarily in major organs, peaking at 24 h post-injection and gradually declining thereafter, with faint fluorescence still detectable in the kidneys after one week. Importantly, the DN group exhibited higher fluorescence intensity in the kidneys compared to normal control, indicating enhanced homing and retention of hUC-MSCs to injured renal tissue. This is consistent with previous reports that MSCs can migrate to sites of damage and participate in repair processes via targeted differentiation or immunomodulation [64, 65].

DN involves damage to key components of the glomerular filtration barrier-podocytes, glomerular basement membrane, and endothelial cells-resulting in functional impairment characterized by elevated blood creatinine, urea nitrogen, and proteinuria [66, 67].

We found that transplantation of both hypoxic and normoxic low-passage(P4) hUC-MSCs led to significant and similar improvement of kidney function. Strikingly, hypoxic high-passage(P16) hUC-MSCs provided comparable improvement to low-passage hUC-MSCs, attenuating podocyte injury, interstitial fibrosis and other pathological changes, improving serum renal damage indicators and reducing proteinuria levels. By contrast, normoxic high-passage (P16) hUC-MSCs showed almost no therapeutic effect.

In DN, elevated cytokine levels such as IL-6, IL-8, TNF-α, and TGF-β drive inflammatory cell infiltration and interstitial fibrosis. TGF-β, in particular, induces early renal hypertrophy by disrupting the cell cycle and promotes interstitial fibrosis via epithelial-to-myofibroblast transition [68, 69]. Our data demonstrate that hypoxic but not normoxic high-passage hUC-MSCs significantly reduced TGF-β levels and downregulated the expression of myofibroblast markers α-SMA and type III collagen in DN mice renal tissues.

In renal RNA sequencing analysis, compared to the 20%O_2_ group, treatment with high-passage hUC-MSCs cultured under 5%O_2_ condition led to significant downregulation of inflammatory factors (e.g., *Tnfsf10*) and fibrosis-related markers (e.g., *Tgf-β*, *Col3a1*). Concurrently, key pathways mediating renal inflammation and fibrosis, TGF-β and Nf-κB were suppressed, along with reduced expression of cytoskeletal proteins and fibronectin.

Collectively, our in vivo results demonstrate that hypoxic culture effectively preserves therapeutic efficacy of high-passage hUC-MSCs. Their potent anti-inflammatory, anti-fibrotic, and glomerular-protective functions appear to be primarily attributed to paracrine mechanisms. Further studies are warranted to elucidate the specific roles and mechanisms of endocrine and paracrine actions mediated by these factors in the context of DN under hypoxic conditions.

## Conclusion

Our study demonstrates that hypoxic culture conditions during in vitro expansion significantly delay cellular senescence and maintain the biological and reparative functions of hUC-MSCs compared to normoxic culture. This work is the first provision of in vivo evidence confirming that high-passage hUC-MSCs cultured under hypoxia retain considerable therapeutic efficacy, supporting their potential for clinical application even after extended serial passaging. These findings offer novel insights for improving the large-scale production of functional hUC-MSCs as off-the-shelf regenerative therapeutics. Further studies are needed to elucidate the precise anti-inflammatory and anti-apoptotic mechanisms through which hypoxia mitigates cellular aging.

## Supplementary Information

**Supplemental Figure** **1. **Characterization of hUC-MSCs: multilineage differentiation potential and surface marker profile. (A) Multilineage differentiation capacity detected by cellular staining. Top: Adipogenic differentiation (Oil Red O staining of lipid droplets, red), Bottom:Osteogenic differentiation (Alizarin Red S staining of calcium deposits, red). (B) Flow cytometry analysis of surface markers: positive markers (≥95%):CD29, CD44, CD73, CD90, CD105; negative markers (≤5%): CD31, CD34, HLA-DR; Isotype controls:IgG1κ (FITC/PE). Scale bars: 100μm (A).

**Supplemental Figure** **2. **Cell size distribution of hUC-MSCs under normoxic and hypoxic conditions during serial passaging. Representative flow cytometry histograms of forward scatter (FSC) intensity across passages(P4, P6, P8, P10, P12, P14, P16, P18). Top: Hypoxia (5%O_2_), bottom: Normoxia (20%O_2_).

**Supplemental Figure** **3. **Validation of DN mouse model and therapeutic monitoring. (A) Dynamic blood glucose levels (mmol/L) during 15-week modeling. (B) Body weight (g) progression during the experimental period. (C) Cr (μmol/L) at endpoint (week 15). (D) BUN (mmol/L) at week 15. (E) 24-hour urinary protein excretion (mg/24h) at week 15. Experimental groups: Control (Healthy mice); DN (Diabetic nephropathy model); hUC-MSCs (DN mice treated with low-passage hUC-MSCs) N = 4 (per group), data are presented as mean ± SEM. *p<0.05, **p<0.01, ***p<0.001, ****p<0.0001 hUC-MSCs *vs*. DN group; ^#^*p*< 0.05, ^####^*p*<0.0001 control *vs.* DN group.

**Supplemental Figure** **4. ***In vivo* biodistribution of hUC-MSCs in DN mice and kidneys. (A) Whole-body fluorescence imaging of CM-Dil-labeled hUC-MSCs at 8, 24, 48 hours, 4 and 7 days post-intravenous infusion. ROI indicates the average fluorescence intensity. (B,C) Quantification of of the average fluorescence intensity of CIL in whole body of DN mice over the week and in the kidneys of DN mice. Groups: DN: Diabetic nephropathy mice receiving CM-Dil-labeled hUC-MSCs, Control: Healthy mice receiving CM-Dil-labeled hUC-MSCs. N = 3 (per group), data=mean ± SEM. ****p< 0.0001 compared to the 24 h group; ^#^*p*< 0.05 compared to the DN group.

## Data Availability

All data generated or analyzed in this study are included in this published article. Any other data will be available on reasonable request from corresponding author.

## Abbreviation

MSCs: Mesenchymal stem cells
hUC-MSs: Human umbilical cord derived-mesenchymal stem cells
MEF: mouse embryonic fibroblasts
DN: Diabetic nephropathy
ROS: Reactive oxygen species
SA-β-gal: Senescence associated β-galactosidase
SPF: Specific pathogen-free
HFD: High-fat diet
STZ: Streptozotocin
HE: Hematoxylin and eosin
PVDF: Polyvinylidene difluoride
SASP: Senescence-associated secretory phenotype
RNA-Seq: RNA sequencing
PPI: Protein–protein interaction
Cr: Serum creatinine
BUN: Blood urea nitrogen
GBM: Glomerular basement membrane

## References

[1] Friedenstein AJ, Petrakova KV, Kurolesova AI, Frolova GP. Heterotopic of bone marrow. Analysis of precursor cells for osteogenic and hematopoietic tissues. Transplantation. 1968;6(2):230–47. 5654088

[2] Hass R, Kasper C, Böhm S, Jacobs R. Different populations and sources of human mesenchymal stem cells (MSC): a comparison of adult and neonatal tissue-derived MSC. Cell Commun Signal. 2011;9:12. 21569606 10.1186/1478-811X-9-12 PMC3117820

[3] da Silva Meirelles L, Chagastelles PC, Nardi NB. Mesenchymal stem cells reside in virtually all post-natal organs and tissues. J Cell Sci. 2006;119(Pt 11):2204–13. 16684817 10.1242/jcs.02932

[4] Le Blanc K, Tammik L, Sundberg B, Haynesworth SE, Ringdén O. Mesenchymal stem cells inhibit and stimulate mixed lymphocyte cultures and mitogenic responses independently of the major histocompatibility complex. Scand J Immunol. 2003;57(1):11–20. 12542793 10.1046/j.1365-3083.2003.01176.x

[5] Liang X, Ding Y, Zhang Y, Tse HF, Lian Q. Paracrine mechanisms of mesenchymal stem cell-based therapy: current status and perspectives. Cell Transplant. 2014;23(9):1045–59. 23676629 10.3727/096368913X667709

[6] Al-Azab M, Safi M, Idiiatullina E, Al-Shaebi F, Zaky MY. Aging of mesenchymal stem cell: machinery, markers, and strategies of fighting. Cell Mol Biol Lett. 2022;27(1):69. 35986247 10.1186/s11658-022-00366-0 PMC9388978

[7] Herranz N, Gil J. Mechanisms and functions of cellular senescence. J Clin Invest. 2018;128(4):1238–46. 29608137 10.1172/JCI95148 PMC5873888

[8] McHugh D, Gil J. Senescence and aging: causes, consequences, and therapeutic avenues. J Cell Biol. 2018;217(1):65–77. 29114066 10.1083/jcb.201708092 PMC5748990

[9] Zhou X, Hong Y, Zhang H, Li X. Mesenchymal stem cell senescence and rejuvenation: current status and challenges. Front Cell Dev Biol. 2020;8:364. 32582691 10.3389/fcell.2020.00364 PMC7283395

[10] Zhao Q, Zhang L, Wei Y, Yu H, Zou L, Huo J, et al. Systematic comparison of hUC-MSCs at various passages reveals the variations of signatures and therapeutic effect on acute graft-versus-host disease. Stem Cell Res Ther. 2019;10(1):354. 31779707 10.1186/s13287-019-1478-4 PMC6883552

[11] Jiang T, Xu G, Wang Q, Yang L, Zheng L, Zhao J, et al. Correction: in vitro expansion impaired the stemness of early passage mesenchymal stem cells for treatment of cartilage defects. Cell Death Dis. 2019;10(10):716. 31558696 10.1038/s41419-019-1939-9 PMC6763441

[12] Carrel A. On the permanent life of tissues outside of the organism. J Exp Med. 1912;15(5):516–28. 19867545 10.1084/jem.15.5.516 PMC2124948

[13] Ejtehadifar M, Shamsasenjan K, Movassaghpour A, Akbarzadehlaleh P, Dehdilani N, Abbasi P, et al. The effect of hypoxia on mesenchymal stem cell biology. Adv Pharm Bull. 2015;5(2):141–9. 26236651 10.15171/apb.2015.021 PMC4517092

[14] Jung Y, Spencer JA, Raphael AP, Wu JW, Alt C, Runnels JR, et al. Intravital imaging of mouse bone marrow: hemodynamics and vascular permeability. Methods Mol Biol. 2018;1763:11–22. 29476484 10.1007/978-1-4939-7762-8_2

[15] Parekkadan B, Milwid JM. Mesenchymal stem cells as therapeutics. Annu Rev Biomed Eng. 2010;12:87–117. 20415588 10.1146/annurev-bioeng-070909-105309 PMC3759519

[16] Antoniou ES, Sund S, Homsi EN, Challenger LF, Rameshwar P. A theoretical simulation of hematopoietic stem cells during oxygen fluctuations: prediction of bone marrow responses during hemorrhagic shock. Shock. 2004;22(5):415–22. 15489633 10.1097/01.shk.0000142185.88094.88

[17] Grayson WL, Zhao F, Bunnell B, Ma T. Hypoxia enhances proliferation and tissue formation of human mesenchymal stem cells. Biochem Biophys Res Commun. 2007;358(3):948–53. 17521616 10.1016/j.bbrc.2007.05.054

[18] Hung SP, Ho JH, Shih YR, Lo T, Lee OK. Hypoxia promotes proliferation and osteogenic differentiation potentials of human mesenchymal stem cells. J Orthop Res. 2012;30(2):260–6. 21809383 10.1002/jor.21517

[19] Holzwarth C, Vaegler M, Gieseke F, Pfister SM, Handgretinger R, Kerst G, et al. Low physiologic oxygen tensions reduce proliferation and differentiation of human multipotent mesenchymal stromal cells. BMC Cell Biol. 2010;11:11. 20109207 10.1186/1471-2121-11-11 PMC2827377

[20] Kim DS, Ko YJ, Lee MW, Park HJ, Park YJ, Kim DI, et al. Effect of low oxygen tension on the biological characteristics of human bone marrow mesenchymal stem cells. Cell Stress Chaperones. 2016;21(6):1089–99. 27565660 10.1007/s12192-016-0733-1 PMC5083677

[21] Efimenko A, Starostina E, Kalinina N, Stolzing A. Angiogenic properties of aged adipose derived mesenchymal stem cells after hypoxic conditioning. J Transl Med. 2011;9:10. 21244679 10.1186/1479-5876-9-10 PMC3033332

[22] Rosová I, Dao M, Capoccia B, Link D, Nolta JA. Hypoxic preconditioning results in increased motility and improved therapeutic potential of human mesenchymal stem cells. Stem Cells. 2008;26(8):2173–82. 18511601 10.1634/stemcells.2007-1104 PMC3017477

[23] Casciaro F, Borghesan M, Beretti F, Zavatti M, Bertucci E, Follo MY, et al. Prolonged hypoxia delays aging and preserves functionality of human amniotic fluid stem cells. Mech Ageing Dev. 2020;191:111328. 32800796 10.1016/j.mad.2020.111328

[24] Ratushnyy A, Lobanova M, Buravkova LB. Expansion of adipose tissue-derived stromal cells at “physiologic” hypoxia attenuates replicative senescence. Cell Biochem Funct. 2017;35(4):232–43. 28589682 10.1002/cbf.3267

[25] Cawthon RM. Telomere length measurement by a novel monochrome multiplex quantitative PCR method. Nucleic Acids Res. 2009;37(3):e21. 19129229 10.1093/nar/gkn1027 PMC2647324

[26] Kajiwara K, Sawa Y. Overexpression of SGLT2 in the kidney of a *P. gingivalis* LPS-induced diabetic nephropathy mouse model. BMC Nephrol. 2021;22(1):287. 34425759 10.1186/s12882-021-02506-8 PMC8383391

[27] Yang YK, Ogando CR, Wang See C, Chang TY, Barabino GA. Changes in phenotype and differentiation potential of human mesenchymal stem cells aging in vitro. Stem Cell Res Ther. 2018;9(1):131. 29751774 10.1186/s13287-018-0876-3 PMC5948736

[28] Hoang DM, Pham PT, Bach TQ, Ngo A, Nguyen QT, Phan T, et al. Stem cell-based therapy for human diseases. Signal Transduct Target Ther. 2022;7(1):272. 35933430 10.1038/s41392-022-01134-4 PMC9357075

[29] Fernie AR, Carrari F, Sweetlove LJ. Respiratory metabolism: glycolysis, the TCA cycle and mitochondrial electron transport. Curr Opin Plant Biol. 2004;7(3):254–61. 15134745 10.1016/j.pbi.2004.03.007

[30] Obradovic H, Krstic J, Trivanovic D, Mojsilovic S, Okic I, Kukolj T, et al. Improving stemness and functional features of mesenchymal stem cells from Wharton’s jelly of a human umbilical cord by mimicking the native, low oxygen stem cell niche. Placenta. 2019;82:25–34. 31174623 10.1016/j.placenta.2019.05.005

[31] Bi B, Schmitt R, Israilova M, Nishio H, Cantley LG. Stromal cells protect against acute tubular injury via an endocrine effect. J Am Soc Nephrol. 2007;18(9):2486–96. 17656474 10.1681/ASN.2007020140

[32] Chacko SM, Ahmed S, Selvendiran K, Kuppusamy ML, Khan M, Kuppusamy P. Hypoxic preconditioning induces the expression of prosurvival and proangiogenic markers in mesenchymal stem cells. Am J Physiol Cell Physiol. 2010;299(6):C1562–70. 20861473 10.1152/ajpcell.00221.2010 PMC3006322

[33] Hu Z, Wang D, Gong J, Li Y, Ma Z, Luo T, et al. MSCs deliver hypoxia-treated mitochondria reprogramming acinar metabolism to alleviate severe acute pancreatitis injury. Adv Sci. 2023;10(25):e2207691. 10.1002/advs.202207691 PMC10477874 37409821

[34] Hayflick L, Moorhead PS. The serial cultivation of human diploid cell strains. Exp Cell Res. 1961;25:585–621. 13905658 10.1016/0014-4827(61)90192-6

[35] Neurohr GE, Terry RL, Lengefeld J, Bonney M, Brittingham GP, Moretto F, et al. Excessive cell growth causes cytoplasm dilution and contributes to senescence. Cell. 2019;176(5):1083-97.e18. 30739799 10.1016/j.cell.2019.01.018 PMC6386581

[36] Dimri GP, Lee X, Basile G, Acosta M, Scott G, Roskelley C, et al. A biomarker that identifies senescent human cells in culture and in aging skin in vivo. Proc Natl Acad Sci U S A. 1995;92(20):9363–7. 7568133 10.1073/pnas.92.20.9363 PMC40985

[37] Birch J, Gil J. Senescence and the SASP: many therapeutic avenues. Genes Dev. 2020;34(23–24):1565–76. 33262144 10.1101/gad.343129.120 PMC7706700

[38] Panda AC, Abdelmohsen K, Gorospe M. SASP regulation by noncoding RNA. Mech Ageing Dev. 2017;168:37–43. 28502821 10.1016/j.mad.2017.05.004 PMC5681880

[39] Weng Z, Wang Y, Ouchi T, Liu H, Qiao X, Wu C, et al. Mesenchymal stem/stromal cell senescence: hallmarks, mechanisms, and combating strategies. Stem Cells Transl Med. 2022;11(4):356–71. 35485439 10.1093/stcltm/szac004 PMC9052415

[40] Pezzi A, Amorin B, Laureano Á, Valim V, Dahmer A, Zambonato B, et al. Effects of hypoxia in long-term in vitro expansion of human bone marrow derived mesenchymal stem cells. J Cell Biochem. 2017;118(10):3072–9. 28240374 10.1002/jcb.25953

[41] Blackburn EH, Epel ES, Lin J. Human telomere biology: a contributory and interactive factor in aging, disease risks, and protection. Science. 2015;350(6265):1193–8. 26785477 10.1126/science.aab3389

[42] Collins K, Mitchell JR. Telomerase in the human organism. Oncogene. 2002;21(4):564–79. 11850781 10.1038/sj.onc.1205083

[43] De Vusser K, Martens D, Lerut E, Kuypers D, Nawrot TS, Naesens M. Replicative senescence and arteriosclerosis after kidney transplantation. Nephrol Dial Transplant. 2020;35(11):1984–95. 33067610 10.1093/ndt/gfaa151

[44] Estrada JC, Albo C, Benguría A, Dopazo A, López-Romero P, Carrera-Quintanar L, et al. Culture of human mesenchymal stem cells at low oxygen tension improves growth and genetic stability by activating glycolysis. Cell Death Differ. 2012;19(5):743–55. 22139129 10.1038/cdd.2011.172 PMC3321628

[45] Zimmermann S, Voss M, Kaiser S, Kapp U, Waller CF, Martens UM. Lack of telomerase activity in human mesenchymal stem cells. Leukemia. 2003;17(6):1146–9. 12764382 10.1038/sj.leu.2402962

[46] Nishi H, Nakada T, Kyo S, Inoue M, Shay JW, Isaka K. Hypoxia-inducible factor 1 mediates upregulation of telomerase (hTERT). Mol Cell Biol. 2004;24(13):6076–83. 15199161 10.1128/MCB.24.13.6076-6083.2004 PMC480902

[47] Anderson CJ, Hoare SF, Ashcroft M, Bilsland AE, Keith WN. Hypoxic regulation of telomerase gene expression by transcriptional and post-transcriptional mechanisms. Oncogene. 2006;25(1):61–9. 16170363 10.1038/sj.onc.1209011

[48] Nimsanor N, Phetfong J, Kitiyanant N, Kamprom W, Supokawej A. Overexpression of anti-fibrotic factors ameliorates anti-fibrotic properties of Wharton’s jelly derived mesenchymal stem cells under oxidative damage. Biosci Trends. 2019;13(5):411–22. 31656260 10.5582/bst.2019.01191

[49] Prabhakar O. Cerebroprotective effect of resveratrol through antioxidant and anti-inflammatory effects in diabetic rats. Naunyn Schmiedebergs Arch Pharmacol. 2013;386(8):705–10. 23612842 10.1007/s00210-013-0871-2

[50] Marinho HS, Real C, Cyrne L, Soares H, Antunes F. Hydrogen peroxide sensing, signaling and regulation of transcription factors. Redox Biol. 2014;2:535–62. 24634836 10.1016/j.redox.2014.02.006 PMC3953959

[51] Li N, Karin M. Is NF-kappaB the sensor of oxidative stress. FASEB J. 1999;13(10):1137–43. 10385605

[52] Charras A, Arvaniti P, Le Dantec C, Dalekos GN, Zachou K, Bordron A, et al. JAK inhibitors and oxidative stress control. Front Immunol. 2019;10:2814. 31867003 10.3389/fimmu.2019.02814 PMC6908489

[53] Bae SH, Sung SH, Oh SY, Lim JM, Lee SK, Park YN, et al. Sestrins activate Nrf2 by promoting p62-dependent autophagic degradation of Keap1 and prevent oxidative liver damage. Cell Metab. 2013;17(1):73–84. 23274085 10.1016/j.cmet.2012.12.002

[54] González P, Lozano P, Ros G, Solano F. Hyperglycemia and oxidative stress: an integral, updated and critical overview of their metabolic interconnections. Int J Mol Sci. 2023. 10.3390/ijms24119352. 37298303 10.3390/ijms24119352 PMC10253853

[55] Wang M, Xiao Y, Miao J, Zhang X, Liu M, Zhu L, et al. Oxidative stress and inflammation: drivers of tumorigenesis and therapeutic opportunities. Antioxidants (Basel). 2025. 10.3390/antiox14060735. 40563367 10.3390/antiox14060735 PMC12189506

[56] Michalak KP, Michalak AZ. Understanding chronic inflammation: couplings between cytokines, ROS, NO, Cai 2+, HIF-1α, Nrf2 and autophagy. Front Immunol. 2025;16:1558263. 40264757 10.3389/fimmu.2025.1558263 PMC12012389

[57] Yu H, Lin L, Zhang Z, Zhang H, Hu H. Targeting NF-κB pathway for the therapy of diseases: mechanism and clinical study. Signal Transduct Target Ther. 2020;5(1):209. 32958760 10.1038/s41392-020-00312-6 PMC7506548

[58] Massagué J, Sheppard D. TGF-β signaling in health and disease. Cell. 2023;186(19):4007–37. 37714133 10.1016/j.cell.2023.07.036 PMC10772989

[59] Wu W, Wang X, Yu X, Lan HY. Smad3 signatures in renal inflammation and fibrosis. Int J Biol Sci. 2022;18(7):2795–806. 35541902 10.7150/ijbs.71595 PMC9066101

[60] Tuttle KR, Bakris GL, Bilous RW, Chiang JL, de Boer IH, Goldstein-Fuchs J, et al. Diabetic kidney disease: a report from an ADA Consensus Conference. Diabetes Care. 2014;37(10):2864–83. 25249672 10.2337/dc14-1296 PMC4170131

[61] Brito AKdS, Mendes AVdS, Acha BT, Oliveira ASdSS, Macedo JL, Cruzio AS, et al. Experimental models of Type 2 Diabetes Mellitus induced by combining hyperlipidemic diet (HFD) and streptozotocin administration in rats: an integrative review. Biomedicines. 2025. 10.3390/biomedicines13051158. 40426986 10.3390/biomedicines13051158 PMC12108808

[62] Hao L, Fang Q, Shanshan Z, Jing W, Sen L, Xinyuan G, et al. Construction and evaluation of STZ-induced diabetes and diabetic kidney disease models in C57BL/6J mice. Front Endocrinol (Lausanne). 2025;16:1711035. 41480368 10.3389/fendo.2025.1711035 PMC12753433

[63] Ezquer ME, Ezquer FE, Arango-Rodríguez ML, Conget PA. MSC transplantation: a promising therapeutic strategy to manage the onset and progression of diabetic nephropathy. Biol Res. 2012;45(3):289–96. 23283438 10.4067/S0716-97602012000300010

[64] Meirelles LS, Nardi NB. Methodology, biology and clinical applications of mesenchymal stem cells. Front Biosci (Landmark Ed). 2009;14(11):4281–98. 19273350 10.2741/3528

[65] Karp JM, Leng Teo GS. Mesenchymal stem cell homing: the devil is in the details. Cell Stem Cell. 2009;4(3):206–16. 19265660 10.1016/j.stem.2009.02.001

[66] Thomas MC. Pathogenesis and progression of proteinuria. Contrib Nephrol. 2011;170:48–56. 21659757 10.1159/000324943

[67] Durvasula RV, Shankland SJ. Podocyte injury and targeting therapy: an update. Curr Opin Nephrol Hypertens. 2006;15(1):1–7. 16340659 10.1097/01.mnh.0000199012.79670.0b

[68] Ina K, Kitamura H, Tatsukawa S, Takayama T, Fujikura Y, Shimada T. Transformation of interstitial fibroblasts and tubulointerstitial fibrosis in diabetic nephropathy. Med Electron Microsc. 2002;35(2):87–95. 12181650 10.1007/s007950200011

[69] Li JH, Zhu HJ, Huang XR, Lai KN, Johnson RJ, Lan HY. Smad7 inhibits fibrotic effect of TGF-Beta on renal tubular epithelial cells by blocking Smad2 activation. J Am Soc Nephrol. 2002;13(6):1464–72. 12039975 10.1097/01.asn.0000014252.37680.e4

